# Rising prevalence of multiple sclerosis in Saudi Arabia, a descriptive study

**DOI:** 10.1186/s12883-020-1629-3

**Published:** 2020-02-08

**Authors:** Mohammed AlJumah, R. Bunyan, H. Al Otaibi, G. Al Towaijri, A. Karim, Y. Al Malik, M. Kalakatawi, S. Alrajeh, M. Al Mejally, H. Algahtani, A. Almubarak, E. Cupler, S. Alawi, S. Qureshi, S. Nahrir, A. Almalki, A. Alhazzani, I. Althubaiti, N. Alzahrani, E. Mohamednour, J. Saeedi, S. Ishak, H. Almudaiheem, A. El-Metwally, A. Al-Jedai

**Affiliations:** 1 King Fahd Medical City (KFMC), MOH, Riyadh, Saudi Arabia; 2 King Fahd Specialist Hospital (KFSH)-Dammam, Dammam, Saudi Arabia; 3 King Fahd General Hospital-Jeddah, Jeddah, Saudi Arabia; 4 King Fahd General Hospital-Al-Madinah, Riyadh, Saudi Arabia; 5 King Abdulaziz Medical City (National Guard Health Affairs)-Riyadh, Riyadh, Saudi Arabia; 6 King Saud bin Abdulaziz University For Health Sciences, Riyadh, Saudi Arabia; 7 Nour Specialized Hospital, Makkah, Saudi Arabia; 8 Dr. Sulaiman Al Habib Hospital-Olaya Branch, Riyadh, Saudi Arabia; 9 Heraa General Hospital, Makkah, Saudi Arabia; 10 King Abdul-Aziz Medical City (National Guard Health Affairs), Jeddah, Saudi Arabia; 11 Qatif Central Hospital, Qatif, Saudi Arabia; 12 King Faisal Specialist Hospital & Research Center, Jeddah, Saudi Arabia; 13 Prince Sultan Military Medical City, Riyadh, Saudi Arabia; 14 Johns Hopkins Aramco Healthcare Company (JHAH), Dhahran, Saudi Arabia; 15 King Saud Medical City, Riyadh, Saudi Arabia; 16 King Abdul-Aziz Hospital and Oncology Center, Jeddah, Saudi Arabia; 17 King Saud University, Riyadh, Saudi Arabia; 18 King Fahad Military Medical Complex, Dhahran, Saudi Arabia; 19 King Fahd General Hospital, Baha, Saudi Arabia; 20 King Fahad Specialist Hospital, Dammam, Qassim Saudi Arabia; 21 King Abdullah Bin Abdulaziz University Hospital, Riyadh, Saudi Arabia; 22 Itkan Health Consulting, Riyadh, Saudi Arabia; 23 Ministry of Health, Deputyship of Therapeutic Affairs, Riyadh, Saudi Arabia

**Keywords:** Prevalence, Multiple sclerosis, Registry, Saudi Arabia

## Abstract

**Background:**

In 2015, the first nationwide, multicenter Multiple Sclerosis (MS) registry was initiated in the Kingdom of Saudi Arabia (KSA) mainly with an objective to describe current epidemiology, disease patterns, and clinical characteristics of MS in Saudi Arabia. This article aimed to report initial findings of the registry and regional prevalence of MS.

**Method:**

In 2015, a national MS registry was launched in KSA to register all MS patient with confirmed diagnosis according to the 2010 McDonald Criteria. The registry aimed to identify and recruit all healthcare facilities treating MS patients in the Kingdom, and collect data such as demographics, clinical characteristics (disease onset, diagnosis, presentation of symptoms at onset, disease course, relapse rate, and disability measures), family history, and treatments. All the included sites have obtained IRB/EC approvals for participating in the registry. Currently, the registry includes 20 hospitals from different regions across the Kingdom. The Projected prevalence was calculated based on the assumption that the number of diagnosed MS cases in participating hospitals (in each region) is similar to the number of cases in remaining nonparticipant hospitals in the same region.

**Results:**

As of September 2018, the registry has included 20 hospitals from the different regions across the Kingdom and has collected comprehensive data on 2516 patients from those hospitals, with median age 32 (Range: 11–63) and 66.5% being females. The reported prevalence of MS for those hospitals was estimated to be 7.70/100,000 population and 11.80/100,000 Saudi nationals. Based on the assumption made earlier, we projected the prevalence for each region and for the country as a whole. The overall prevalence of MS at the country level was reported to be 40.40/100,000 total population and 61.95/100,000 Saudi nationals. Around 3 out of every 4 patients (77.5%) were 40 years of age or younger. Female to male ratio was 2:1. The prevalence was higher among females, young and educated individuals across all five regions of Saudi Arabia.

**Conclusion:**

The prevalence of MS has significantly increased in Saudi Arabia but is still much lower than that in the western and other neighboring countries like Kuwait, Qatar, and the UAE. However, compared to the past rates, Saudi Arabia’s projected prevalence of MS through this national study is 40.40/100,000 population, putting the Kingdom above the low risk zone as per Kurtzke classification. The projected prevalence was estimated to be much higher among Saudi nationals (61.95/100,000 Saudi-nationals). The prevalence was higher among female, younger and educated individuals. Further studies are needed to assess the risk factors associated with increased prevalence in Saudi Arabia.

## Background

Multiple sclerosis (MS) is one of the most common inflammatory neurological disorders with the global prevalence of 33/100,000. Prevalence has increased substantially in many regions since 1990, especially in various low and middle income countries across the globe imposing a significant economical and health care burden [1–3]. MS is potentially a progressive disease of central nervous system that leads to life-long disabilities and impacting the productivity of individuals affected and patient and family quality of life. Many patients with multiple sclerosis enter a progressive phase of the disease one to two decades after onset. Common neurological manifestations include vision impairment, loss of co-ordination and balance, fatigue, pain, bladder dysfunction, cognitive dysfunction, numbness, weakness and mood changes [4, 5].

Pathophysiology of MS is complex and is considered as an autoimmune disease triggered by auto-reactive CD4 and helper T cells causing demyelination. However genetic and infectious factors have been reported by many studies [6, 7]. Genetic disposition is also reported for MS, females being more affected compared to males [8]. With respect to age, prevalence of MS increased after adolescence, reaching a peak between 25 and 35 years. Other environmental factors such as vitamin D levels, obesity, and certain early childhood exposure to infectious agents, smoking, and certain other modifiable risk factors are associated with increased risk of MS. [9]

As per Kurtzke classification, prevalence of MS in the Gulf region is marked as low-risk zone, however recent data have shown marked increased in the prevalence of MS, nearly 31–55 MS per 100,0000 individuals [10]. The increased prevalence in the Gulf region could be associated with change in lifestyle conditions, vitamin D deficiency and parental consanguinity as reported by few studies [11, 12]. However, the lack of a central MS registry and long term follow-up epidemiological studies, make it difficult to understand the actual prevalence and associated factors related to the increasing number of MS cases in the region.

Unlike neighboring Gulf countries (ex. UAE, Qatar, & Kuwait), the prevalence of MS was not as high in Saudi Arabia, and MS was not considered as a significant concern in the Kingdom compared to western and other gulf countries, mainly because the lack of data and underreporting of cases [11]. In the period of 2015–2018, the first nationwide, multicenter MS registry was initiated in the Kingdom mainly with the objective to describe current epidemiology, disease patterns, and clinical characteristics of MS in Saudi Arabia. The data reported increased prevalence of MS in the Kingdom, which is alarming and warrants an immediate public health action [13].

This article aimed to report initial findings of the national registry regarding the prevalence of MS, which in turn would be a valuable repository of information on the characteristics and long-term course of MS in the Kingdom. Reporting the actual burden of MS by using these cross sectional and longitudinal databases aim to offer significant information that can promote a better understanding of the prognosis and risk-factors, as well as serve as an essential guide for both socioeconomic and clinical decision-making. It offers insights into the progression and process of the disease, together with its effect on the morbidity, quality of life and functional status of the patients. Patients’ registries are, in general, a valuable source of information as they provide significant data about a disease/disorder that cannot be otherwise captured.

## Method

**Fig. 1:**
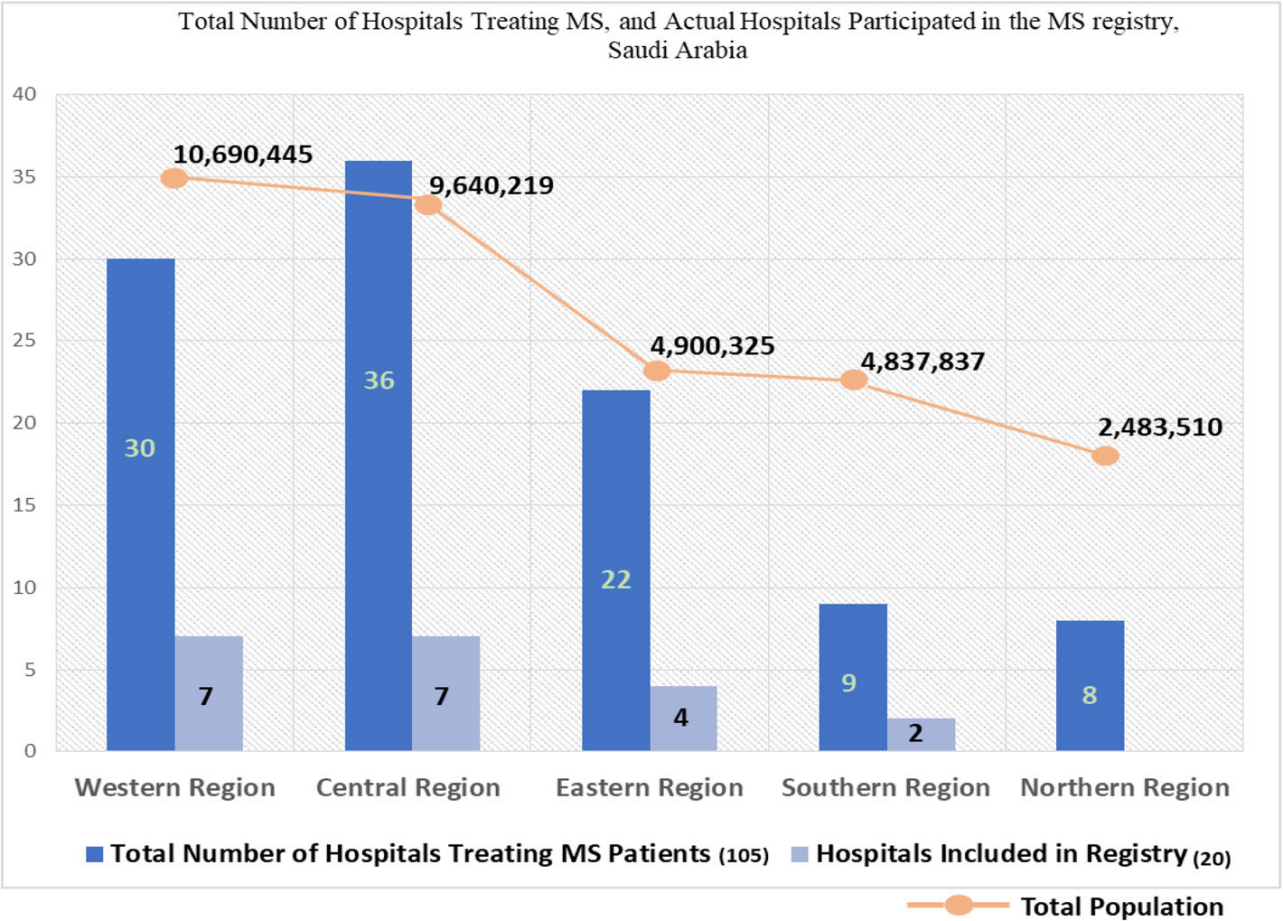
Total population across the Kingdom, Total Number of Hospitals Treating MS, and Actual Hospitals Participated in the MS registry, Saudi Arabia

In 2015, a national MS registry was launched in Saudi Arabia to register all patients with confirmed MS diagnosis according to the 2010 McDonald Criteria. The registry managed by a steering committee (consisting of three main investigators) and a scientific committee (consisting of 10 neurologists from different sites across the different regions in the Kingdom). The study was carried out as a cross-sectional study, with an aim to identify and recruit all healthcare facilities in the Kingdom and collect disease-related data. All the sites included in the registry to date have obtained Institutional Review Board / Ethical Committee approvals for participating in the registry. Currently, 20 hospitals across the kingdom are included in the registry (Fig. 1).

### Data collection

The data was directly entered by participating physicians into a Web-based Application accessible through the internet in participating sites. Variables included in the registry were structured, discussed and approved by the two registry committees and workgroups (Steering and Scientific). Data collected include but not limited to demographics, clinical characteristics (disease onset, diagnosis, presentation of symptoms at onset, disease course, relapse rate, and disability measures), family history, and treatments.

### Data management and cleaning

Validation of different data points was conducted at each of the sites. Missing data were checked, identified with appropriates codes during the analysis and indicated in the tables appropriately. Missing data was categorized for mandatory variables and non-mandatory variables. However, for applying test of significance, missing data was excluded.

### Statistical analysis

The following is a general description of the planned statistical methods used for analysis of the data collected until 2018:

Frequency distribution tables were generated according to the regions. Continuous and discreet quantitative variables (age, time duration, etc.) were summarized in terms of descriptive statistics such as mean (Standard deviation) and Median (Inter quartile range) as per normality test. Categorical variables such as gender, educational status, and marital status were described as the total number and relative percentage of patients per response category. To evaluate the association between different categorical variables, bivariate analyses were also performed. To test for differences in the mean values (when the data followed normal distribution) between different strata, one way analysis of variance (ANOVA) was employed. If the data revealed statistical significance, further inter comparisons amongst the groups was carried out by using post hoc Bonferroni Method. Similarly, to compare the difference in the mean values between different strata, student t-test was employed. Chi square and Fisher exact test were reported for categorical variables. Data analysis was performed with statistics software SPSS 22.0. Statistical significance was set at *p*-value < 0.05. For estimating prevalence, population data of 2017 was used.

The Projected prevalence was calculated based on the assumption that the number of diagnosed MS cases in participating hospitals (in each region) is similar to the number of cases in remaining nonparticipant hospitals in the same region. Accordingly, the total number of patients was then estimated for each individual region and for the country as a whole. Finally, the projected prevalence was estimated at both levels, regional and country level.

Please note that the numerator in all region-specific prevalence calculations were represented by cases residing in that specific region regardless of the hospital’s region (where patients’ were diagnosed).

## Results

As of September 2018, the registry has included 20 hospitals from the different regions across the Kingdom and has collected comprehensive disease-related data on 2516 patients from those hospitals, with median age 32 (Range: 11–63) and 66.5% being female. The reported prevalence of MS in Saudi Arabia in the included hospitals was 7.70/100,000 population (Number of cases in all hospitals included in registry / total Saudi population). Assuming the number of diagnosed MS cases in included hospitals in the registry (in each region) and excluded hospitals (that are capable of diagnosing and treating MS) are the same, we estimated the total number of MS patients across the whole Kingdom and in each region individually. Finally, we estimated the projected prevalence of MS for the kingdom and for each region by dividing the projected number of cases by the total population in Saudi Arabia (for projected country prevalence) or region-specific population (for region-specific projected prevalence). Accordingly, the estimated overall prevalence was found to be 40.4/100,000 population. (Table 1) Prevalence of MS was found to be higher for western region followed by central, eastern, northern and southern region. A different trend of projected prevalence has been noticed with respect to regions, highest for central region followed by eastern, western, northern and southern.

**Table 1:** MS prevalence for Saudi Arabia

Regions	Total hospital (MS)	Hospitals Included in Registry	MS patients from 20 hospital	Prevalence/100000 population	Projected prevalence/100000 population
Western Region	30	7	990	9.3	39.9
Central Region	36	7	866	9.0	46.3
Eastern Region	22	4	378	7.7	42.4
Southern Region	9	2	173	3.6	16.2
Northern Region^a^	8	0	109	4.4	35.2
Total “2017”	105	20	2516	7.7	40.4

^a^Patients referred to other regions. For calculating projected prevalence for northern region we assumed 109 patients were referred from a single hospital of northern region to hospitals in other regions

Among Saudi nationals, the total prevalence of MS was estimated to be 11.80/100,000 per Saudi nationals, and the projected prevalence was estimated to be 61.95/100,000 per Saudi nationals [14]. The prevalence was higher in western region followed by central, eastern, northern and southern regions. However, projected prevalence was estimated to be higher in central region followed by western, eastern, northern and southern regions. (Table 2).

**Table 2:** Prevalence of MS among Saudi Nationals

Regions	Saudi nationals	MS patients from 20 hospital (Saudi Nationals)	Prevalence/ 100,000 population	Projected prevalence/100000 population
Western Region	5,892,821	931	15.8	67.7
Central Region	5,667,865	833	14.7	75.6
Eastern Region	5,892,821	371	7.6	64.9
Southern Region	3,777,879	173	3.6	20.7
Northern Region^*^	1,929,435	107	5.5	44.0
Total “2017”	20,408,362	2415	11.8	61.95

* Patients in the northern region were referred to facilities in other regions. For calculating projected prevalence for northern region we assumed 107 Saudi patients were referred from a single hospital of the northern region to hospitals in other regions

The prevalence of MS was higher among females across all the regions (*p*-value < 0.001 for all regions). Of the total 2516 patients, data per region was available for 2507 patients with some missing information, therefore, the analysis was done on available data only. 77.5% of the patients were 40 years of age or younger and the variance in age was significantly different among the regions with *p* value of 0.006. With respect to the educational level, majority were University graduates and post-graduates (57.4%) followed by high school (31.9%), primary school (9.4%) and illiterate only 1.3%. This trend in educational level was seen among all the regions. With respect to the employment, it was observed that 41.6% of the total cases were employed, 48.2% were unemployed, and 10.2% were students. Of all cases, majority were married (60.0%), followed by singles (36.3%) and 3.6% divorced/widow (er). Level of education and occupation significantly differed across the regions with *p*-value < 0.001for both. (Table 3) Across all the regions, nearly 86.9% of the patients got education material at the time of diagnosis. Among the total referrals, more than half of the patients (54.2%) were referred by Government/public hospitals, private referrals were 28.2%, and personal referrals were the least and made 17.6% of the total. The referral pattern, however, varied between the regions with *p*-value <0.001 for all. The median age of 1st attack was 27 (Range: 1–72) with significant difference between western and eastern and western and central regions with *p*-value of 0.044 and 0.045 respectively. The median number of previous relapse was 1 (Range:0–24). Lowest numbers were reported in the eastern region compared to other regions with *p-*value of <0.001, and also a significant difference was found between the central and southern regions with p-value 0.023. The most common presenting symptom at first attack included muscle weakness (57.1%), followed by visual symptoms (48.2%) and sensory symptoms (47.3%) among all the cases in all the regions. Similar distribution was observed between the regions where majority had muscle weakness followed by visual symptoms. Among the total, majority of MS had undergone MRI investigation (98.4%) followed by Evoked potential (46.9%) and CSF (37.7%). Majority of the patients were diagnosed with relapsing remitting form of MS (92.6%), it varied between the regions from 90.3–94.4%. the Median EDS score was 1 (IQR 0–2.5) indicating that majority had no or minimal disability.

**Table 3:** Distribution of baseline characteristics among the patients across all the regions of KSA (*n* = 2507)

Variable	Total (%)	Western Region (%)	Central Region (%)	Eastern Region (%)	Southern Region (%)	Northern Region (%)
Gender						
Male	839(33.5)	125(33.3)	287(33.3)	325(32.9)	59(34.1)	43(39.4)
Female	1668(66.5)	250(66.7)	575(66.7)	663(67.1)	114(65.9)	66(60.6)
Age^a^						
</=40	1941(77.5)	728(73.8)	698(81.0)	294(78.4)	137(79.2)	84(77.1)
> 40	565(22.5)	259(26.2)	164(19.0)	81(21.6)	36(20.8)	25(22.9)
Education^a^						
Illiterate	32(1.3)	20(2.0)	5(0.6)	4(1.1)	3(1.7)	0(0.0)
Primary	235(9.4)	106(9.4)	68(7.9)	27(7.2)	21(12.1)	13(11.9)
High school	798(31.9)	284(28.8)	257(30.0)	166(44.5)	53(30.6)	38(34.9)
University and above	1434(57.4)	577(58.5)	527(61.5)	176(47.2)	96(55.5)	58(53.2)
Occupation^a^						
Employed	1040(41.6)	380(38.5)	382(44.6)	155(41.4)	72(41.6)	51(47.2)
Unemployed	1203(48.2)	516(52.3)	363(42.4)	189(50.6)	82(47.4)	53(49.1)
Student	56(10.2)	91(9.2)	112(13.1)	30(8.0)	19(11.0)	4(3.7)
Marital status^a^						
Single	910(36.3)	334(33.8)	327(38.0)	146(38.9)	72(41.6)	31(28.4)
Divorced/widow (er)	91(3.6)	35(3.5)	35(4.1)	11(2.9)	5(2.9)	5(4.6)
Married	1504(60.0)	619(62.7)	498(57.9)	218(58.1)	96(55.5)	498(57.9)
Education Material^a^						
Given	1963(86.9)	742(93.8)	645(77.9)	355(95.7)	126(76.8)	95(89.6)
Not Given	297(13.1)	49(6.2)	183(22.1)	16(4.3)	38(23.2)	11(10.4)
Age at 1st attack (Mean SD)	27.82(8.86)	28.50(9.39)	27.29(8.22)	26.93(8.81)	27.54(8.81)	29.50(8.64)
No of previous relapse (Mean SD)	1.10(1.41)	1.20(1.45)	1.05(1.47)	0.70(1.09)	1.41(1.53)	1.32(1.13)

^a^Indicating missing data

## Discussion

Multiple sclerosis (MS) is one of the most commonly found autoimmune diseases that affect the central nervous system (CNS), and tends to affect adults during their most productive years. Generation of a disease registry is a systematic procedure for gathering data related to patients with certain conditions and diagnosis, or individuals who underwent certain procedures to help in tracking clinical care and outcomes. These registries are a valuable source for both, clinicians and policy makers, as they provide real-world evidence on the actual burden of a certain disease, the quality and effectiveness of clinical practices, patient outcomes, and health care services’ utilization. A registry also allows patients’ identification, enhances care coordination and disease management.

Previously, the Gulf region was thought to have a low MS prevalence, but in the last few years, data have shown a significant increase in the number of MS cases in the region in general, including Saudi Arabia, with prevalence rates ≥30/100,000. There are still no National or regional registries in the region to help understand the actual burden of the disease and reach an accurate estimate of the prevalence. In 2015, the first nationwide, multicenter MS registry was initiated in Saudi Arabia with the objective to describe current epidemiology, disease patterns, and clinical characteristics of MS in the Kingdom. Through this article, we reflected the current prevalence of MS which was estimated at 7.70/100,000 population based on the registry data from the 20 hospitals included. Accordingly, the overall prevalence at the total population (country) level was then projected, and was estimated at 40.40/100,000 population. The prevalence was estimated to be the highest for the central region, followed by the eastern, western, northern and southern regions. Moreover, prevalence was higher among females, young, educated, employed and married individuals across the western, eastern, central, southern and northern regions of the Kingdom.

It is noteworthy to point out that the difference in MS prevalence between the regions in the Kingdom maybe attributed to a number of factors, one of which is the differences in the demographic characteristics between the regions. For example, in the Southern region, the young population aged 19 years or younger represent 36% of the total region’s population compared to 24% of the Western region’s population, hence the lower prevalence in the South [14]. In addition, the number of referral hospitals and neurologists are much smaller in the Southern region compared to the other regions.

The estimated prevalence in Saudi Arabia was lower than that reported in studies conducted in Kuwait (104.88 / 100,000 persons), Qatar (64.57 / 100, 000), UAE (57.09 / 100,000) and Iran (54.51 / 100,000). However, the prevalence among Saudi nationals only was comparable to the prevalence reported in the above-mentioned studies [15–18]. Moreover, when compared to other western regions, the prevalence in Saudi Arabia is considered low. The prevalence in other western countries was reported as high as 98.4/100,000, 106.6/100,000, 170.9/100,000 and 179.9/100,000 in central Italy, Italy, France, Columbia and Canada respectively [19–22]. The variation in the prevalence could be related to many factors, for example genetic variation, certain environmental factors, role of ethnicity and other variations related to methodologies followed in the different studies; however, the increased prevalence is consistently reported across the different studies conducted globally and in neighboring countries. The female to male ratio in our study was 2:1 nearly similar to the two recent studies conducted in the MENA region, R. Alroughani et al. and Hamdy et al., where the female to male ratio was reported at 1.84:1 and 2.14: 1 respectively [15, 23]. The mean age at disease onset was 27.8 years, similar to the reported in the overall estimate in a meta-analysis of 52 studies in the MENA region of 28.54 years (27.61–29.48 years) [18].

The prevalence reported in this paper should be interpreted cautiously as the prevalence has been projected based on the assumption that the number of diagnosed MS cases in included hospitals in the registry (in each region) and excluded hospitals (that are capable of diagnosing MS) in the same region are the same, which could have under or overestimated the projected rate, as the actual number of cases in the hospitals that are not in included the registry are not yet known. However, it must be noted that the registry aimed at including all hospitals and all hospitals were invited to take part in this national effort. We have no evidence to suggest that data from hospitals that actually participated in the registry would significantly differ from those who still did not take part in terms of hospital size or capabilities in diagnosing MS.

This registry was able to collect data from 20 hospitals out of 105 hospitals diagnosing and treating MS population covering only 19% of the total hospitals. Discussions related to the current data of the registry is ongoing with the Ministry of Health (MOH), and work is in progress to lunch a new longitudinal registry by 2020 with the support of MOH involving all hospitals in KSA. Moreover, with the continuous advancements in diagnostic techniques and increased awareness, reporting of MS varies with time and that might affect the true prevalence of MS. However, since the initiation of this registry, the diagnosis of MS has been consistent across the Kingdom, thus it is highly unlikely that the rates of MS have changed significantly in the past few years.

In conclusion, the Saudi Multiple sclerosis registry was able to establish baseline epidemiological data on MS and potentially inform the healthcare providers, healthcare planners, patients and the scientific community. The data obtained from the registry will help as a foundation for understanding and evaluating the current clustering and geography of MS, gender history and variances in the gender ratio of the disorder allowing future healthcare planning and promotion of advocacy, as well as support a wide array of research initiatives.

## Conclusion

This article is the first step toward reporting the current prevalence of MS in Saudi Arabia by reporting the findings of the MS registry. With the available data, the projected prevalence of MS was estimated at 40.40/100,000 total population putting Saudi above the low risk zone as per Kurtzke classification. Furthermore, the projected prevalence was estimated to be much higher among Saudi nationals only at 61.95/100,000 Saudi nationals. Further studies are needed to assess the risk factors associated with the increased prevalence in Saudi Arabia.

## Data Availability

Data and relevant material is available upon request. However, access to the data is restricted to public and require appropriate approvals from the authorized and participating parties.

## Abbreviations

ANOVA: One way analysis of variance
CNS: Central nervous system
KSA: Kingdom of Saudi Arabia
MENA: Middle East and North Africa
MOH: Ministry of Health
MS: Multiple Sclerosis
